# *Surviving & Thriving*; a healthy lifestyle app for new US firefighters: usability and pilot study protocol

**DOI:** 10.3389/fendo.2023.1250041

**Published:** 2023-10-05

**Authors:** Maria Soledad Hershey, Eleni Bouziani, Xin Yu (Maggie) Chen, Irene Lidoriki, Kishor Hadkhale, Ya-Chin Huang, Theodoros Filippou, José Francisco López-Gil, Anne Katherine Gribble, Fan-Yun Lan, Mercedes Sotos-Prieto, Stefanos N. Kales

**Affiliations:** ^1^ Department of Environmental Health, Harvard T.H. Chan School of Public Health, Boston, MA, United States; ^2^ Department of Medicine, School of Medicine, National and Kapodistrian University of Athens, Athens, Greece; ^3^ Harvard Faculty of Arts and Sciences, William James Hall, Cambridge, MA, United States; ^4^ Department of Occupational Medicine, Cambridge Health Alliance, Harvard Medical School, Cambridge, MA, United States; ^5^ Department of Preventive Medicine, Kaohsiung Municipal Ta-Tung Hospital, Kaohsiung Medical University, Kaohsiung, Taiwan; ^6^ Department of Occupational & Environmental Medicine, Kaohsiung Medical University Hospital, Kaohsiung Medical University, Kaohsiung, Taiwan; ^7^ Covid-19 Clinic, Limassol General Hospital, Limassol, Cyprus; ^8^ One Health Research Group, Universidad de Las Américas, Quito, Ecuador; ^9^ Illawarra Shoalhaven Local Health District, NSW Health, Warrawong, NSW, Australia; ^10^ Institute of Health and Welfare Policy, National Yang Ming Chiao Tung University, Taipei, Taiwan; ^11^ Department of Preventive Medicine and Public Health, School of Medicine, Universidad Autónoma de Madrid, and IdiPaz (Instituto de Investigación Sanitaria Hospital Universitario La Paz), Madrid, Spain; ^12^ Center for Biomedical Research in Epidemiology and Public Health Network (CIBERESP) [Consorcio Centro de Investigación Biomédica en Red (CIBER) of Epidemiology and Public Health], Madrid, Spain; ^13^ Madrid Institute for Advanced Studies (IMDEA)-Food Institute, The Campus of International Excellence (CEI), The Spanish National Research Council (CSIC), The Autonomous University of Madrid (UAM), Madrid, Spain

**Keywords:** lifestyle, digital health interventions, health promotion, field-testing, firefighting, workplace interventions, workplace well-being

## Abstract

In the United States (US), new firefighters’ fitness and health behaviors deteriorate rapidly after fire academy graduation. Over the long-term, this increases their risks for chronic diseases. This study protocol describes the proposed usability testing and pilot study of a newly designed and developed healthy lifestyle smartphone app, “Surviving & Thriving”, tailored towards young US firefighters. “Surviving & Thriving” will provide interactive educational content on four lifestyle factors; nutrition, sleep, physical activity, and resilience, and include a personalized journey, habit tracker, and elements of gamification to promote engagement and long-term healthy behavior change. The first phase of the app development entails alpha testing by the research team and pre-beta testing by a fire service expert panel which will help refine the app into a pre-consumer version. Upon completion of the full app prototype, beta ‘usability’ testing will be conducted among new fire academy graduates from two New England fire academies to collect qualitative and quantitative feedback via focus groups and satisfaction surveys, respectively. A last phase of piloting the app will evaluate the app’s efficacy at maintaining/improving healthy lifestyle behaviors, mental health metrics, and physical fitness metrics. We will also evaluate whether firefighters’ perceived “health cultures” scores (ratings of each fire station’s/fire department’s environments as to encouraging/discouraging healthy behaviors) modify the changes in health metrics after utilizing the app for three to six months. This novel user-friendly app seeks to help new firefighters maintain/improve their health and fitness more effectively, reducing their risk of lifestyle-related chronic disease. Firefighters who can establish healthy habits early in their careers are more likely to sustain them throughout their lives.

## 1 Introduction

Cardiovascular diseases (CVD) account for 45% of on-duty deaths among United States (US) firefighters (1, 2). Moreover, leading risk factors for heart disease, such as overweight, obesity and metabolic syndrome, are highly prevalent among firefighters (3, 4). Even among younger firefighters, sudden cardiac death has been associated with preventable risk factors, in particular obesity and hypertension (5). Therefore, the career fire service represents a working population at high risk for obesity, CVD, and cancer (1, 6–8) Nonetheless, evidence has suggested public health strategies targeting firefighters’ lifestyle may help reduce risk factors and prevent the onset of chronic disease and premature death (9–12). A healthy lifestyle (HLS) is an evidence-based synergy of behaviors that has been associated with increased well-being and cardiopulmonary fitness, improved anthropometrics and cardiometabolic parameters, and lower blood pressure among firefighters, decreasing disability and chronic disease in this high CVD risk working population (12, 13).

Health promotion programs have demonstrated positive health effects across diverse workplaces, including the fire service (14, 15). As an exemplary HLS, numerous studies have demonstrated that the traditional Mediterranean lifestyle and diet are effective for reducing cancer death, CVD morbidity and mortality, and all-cause mortality (16, 17). Physical activity interventions have also suggested risk reductions for CVD, workplace injuries and occupational stress, as well as improvements in physical fitness and muscular strength (18–20). Yet, firefighters are often quite sedentary in their workplaces (3), and exercise programs are rarely mandated. In addition, due to the nature of shift work, many firefighters report poor sleep quality, sleep disturbances, and sleep disorders, which have been associated with impaired cardiometabolic and mental health outcomes (21–23). Firefighters are also repeatedly exposed to traumatic events which may pose a significant burden on mental health (24). Nevertheless, preventive strategies for resilience training on mindfulness, emotional intelligence, and stress management for first responders have demonstrated positive health benefits (25).

Career firefighter recruits in the US typically undergo formal training in a designated Fire Academy for 12-20 weeks before becoming new or “probationary” firefighters during their first year of service (26). A recent study demonstrated that fire academy training significantly improved recruits’ body composition, aerobic capacity (1.5-mile run), muscular strength (push-up and pull-up), and muscular endurance (10). However, these fitness and health benefits significantly decreased before these recruits finished their probationary period after academy training. Moreover, firefighters after graduating from the fire academy began to exercise less, eat more poorly, and blood pressure continued to increase despite the health/fitness improvements gained during academy training (7, 24, 27). Nonetheless, a recently implemented HLS intervention in firefighters’ academy training showed improvements in healthy lifestyle behaviors, blood pressure, and mental health (11).

The primary aim of this study is to develop and pilot a user-friendly HLS smartphone application (app) tailored towards new probationary firefighters to consolidate and extend academy/training-derived health benefits into firefighters’ first year in the fire service, and then beyond. The first phase of our research project includes the compiling and designing of educational content for a multicomponent lifestyle intervention addressing physical activity, nutrition, sleep, and resilience, the corresponding app software development, and testing of the minimal viable product (MVP). In the second and third phases of this project, we will test the final HLS app prototype’s user-acceptance and effectiveness. The pilot study will evaluate the app’s ability to engage new firefighters and demonstrate whether adherence to the HLS app-based content is associated with measurable health benefits. New firefighters who can maintain a healthy lifestyle during their initial year in the fire service will be more likely to maintain good health behaviors throughout their careers.

We propose the following three aims for the development, usability testing, and piloting of this novel study intervention tool:

Aim 1- HLS app prototype: Develop a smartphone app for future utilization as a digital HLS intervention directed at new US firefighters. Alpha testing by the study team and pre-beta testing by a fire service expert panel will provide feedback on the MVP to enhance the ‘end-user ready’ *Surviving & Thriving* app.

Aim 2- Usability testing: Conduct beta testing of the HLS app prototype by fire academy graduates working as probationary firefighters among veteran firefighters in typical career fire service environments. This field pilot test includes collecting the users’ qualitative and quantitative feedback regarding user acceptance via focus groups and satisfaction surveys, respectively.

Aim 3- Pilot study: Assess the HLS app for its ability to maintain adherence to the HLS app content for at least 3-6 months and its effects on maintaining/improving HLS parameters (HLS scores, physical fitness, and behavioral health (trauma/depression) screeners) in US career firefighters.

## 2 Materials

### 2.1 Study population & screening procedures

App development, Aim 1, included alpha testing by the research team and pre-beta testing by a fire service expert panel, which helped refine the app into a pre-consumer version. The research team was comprised of health professionals with expertise in lifestyle behaviors (i.e. nutritionists, exercise physiologist, mental health practitioner, and occupational health physician-clinicians) as well as a UX (user experience) and UI (user interface) designer. The Fire Service expert panel included professionals with current or past experience in firefighting and health promotion in the US fire service. For Aims 2 and 3, the target study population of new firefighters will be recruited from the graduated classes of the research team’s partner fire academies in Connecticut and Massachusetts (MA). These firefighting academies have previously committed to participate in the usability testing and pilot study phases. Each academy trains two classes of fire recruits annually, respectively. The academy staff will assist in the recruitment of previous fire academy graduates and provide information about the study without being directly involved in any research activities. Additionally, the academies will hold an online session for further explanation of participants’ involvement and the online informed consent process. Subsequently, the informed consent form will be distributed to eligible academy graduates. Those who provide consent will specify their preferred contact information for future communication with the study team by text or email.

All new firefighters ≥18 years old, English speakers, and who have completed the training at our partners’ training sites (i.e., Connecticut Fire Academy and Massachusetts Firefighting Academy in Stow, MA) will be eligible to participate in the study, either for usability testing or piloting of the final prototype. We anticipate all recruits at these partner academies will be eligible to participate in this research study, requiring no further screening process. For Aim 2, we will recruit recently graduated firefighters from Stow, MA. For Aim 3, we will recruit fire academy graduates from Connecticut’s fire academy. Therefore, participants in the usability testing will differ from the cohort of probationary firefighters in the pilot phase.

### 2.2 HLS app prototype

**Figure 1:**
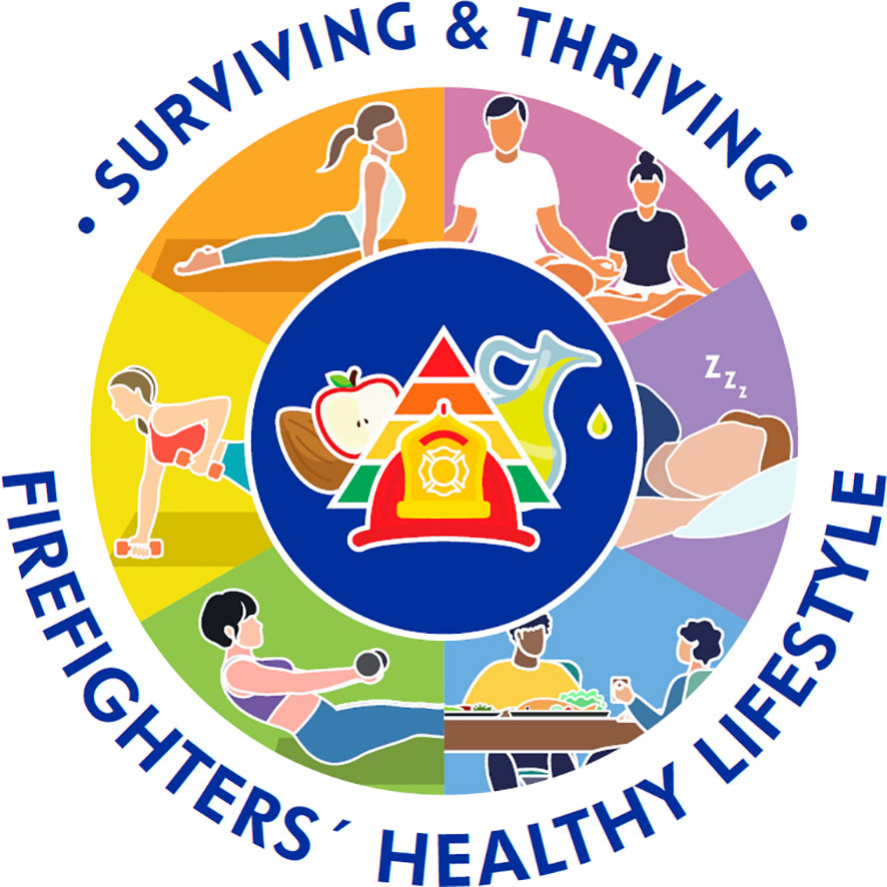
*Surviving & Thriving* mobile application logo.

The HLS app prototype for mobile device platform(s) (IOS/Android) is intended to be used in future HLS interventions directed at new firefighters. Using firefighters’ direct input, existing scientific literature, and reliable online resources, this HLS app titled “Surviving & Thriving” will be designed and developed to help firefighters implement and maintain lifestyle habits that optimize their health and functional performance. The app will promote the combination of balanced nutrition, regular physical activity, restorative sleep, positive social and family connections along with resilience/stress-reduction strategies, and the avoidance of tobacco, binge-drinking, and other toxic substances. In order to accomplish this goal, the app will provide a wide array of multi-domain content (i.e. physical activity, sleep, nutrition, resilience), deliver weekly motivational messages, promote the use of a habit tracker, and facilitate user-interaction through team missions. The content of the HLS app has been visually depicted in our “Surviving and Thriving” app logo (**Figure 1**).

**Figure 2:**
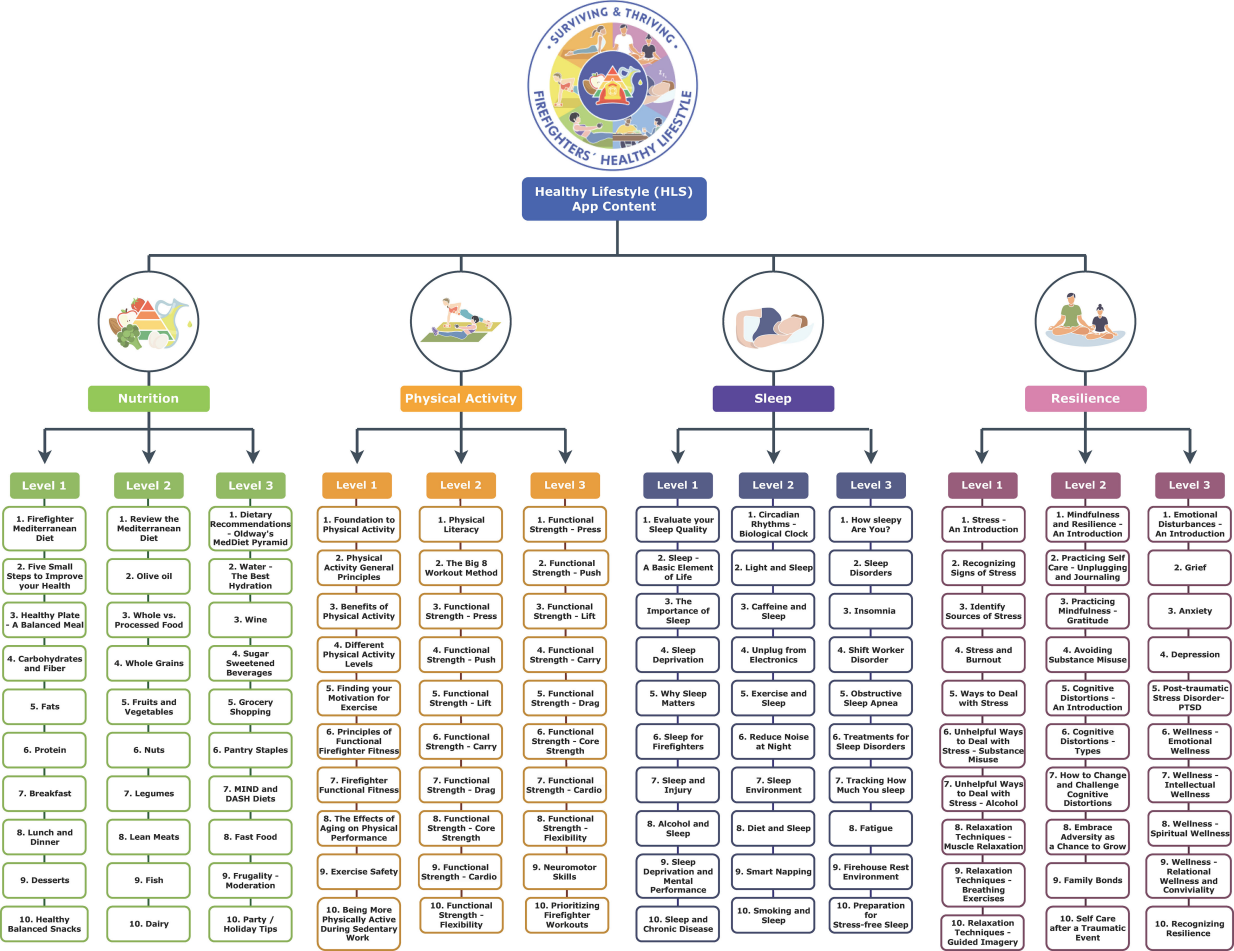
*Surviving & Thriving* healthy lifestyle app content themes across 4 lifestyle domains, 3 levels, 10 quests per level.

**Figure 3:**
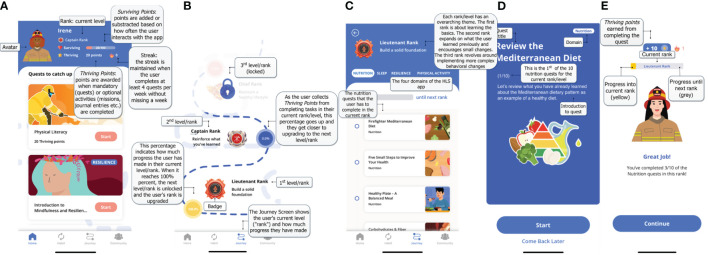
Screenshots of the *Surviving and Thriving* app prototype **(A)** Homepage **(B)** Journey page **(C)** Quest Progress **(D)** Quest Introduction **(E)** Quest Completion.

The final app prototype will include a total of three self-study levels in each HLS domain. Each level is comprised of 10 quests. These levels reflect career firefighter rankings (i.e. Lieutenant, Captain, Chief) and are represented with corresponding badges. Quests are comprised of educational content pertinent to each of the four designated lifestyle factors specifically tailored to the needs of career firefighters (**Figure 2**). The educational component is followed by a corresponding mechanical task that either promotes reinforcement of learned concepts or behavior change that can be recorded in the app’s habit tracker. This evidence-based content was created by the research study team based on information, audiovisual content, and validated assessment tools from various accredited online sources and peer-reviewed articles. To enhance user engagement, the HLS app also utilizes gamification mechanisms that allow users to create personalized firefighter avatars to journey through the three levels of quests and complete missions both individually and as teams of firefighters who can cross-motivate each other. Of note, alpha and pre-beta testing detailed in the following section, will utilize the MVP which is limited to the first level of quests. Operational individual components, namely the educational materials, onboarding survey and personalized journey, *surviving* and *thriving* point system, and gamification mechanisms (e.g. users’ avatar set-up, habit tracker, community and leaderboard, and missions) will then be assembled into a final viable prototype. Detailed wireframes will be made for all the educational material and gamification features by a UI/UX designer with the appropriate design software, Figma (Figma Inc., San Francisco, California USA) (**Figure 3**). The app development with all the necessary platforms and interfaces for both iOS and Android devices will be carried out by a contracted Managed IT Services provider. The app itself is an intervention tool and no identifiable data will be collected directly through the app.

#### 2.2.1 Nutrition domain

The main objective of the Nutrition domain is to help firefighters integrate a healthy balanced diet into their daily living. The first level focuses on the fundamental principles of nutrition (i.e., what are macronutrients, how to make a balanced meal etc.). The second level addresses specific food categories (i.e., whole foods, olive oil, lean protein etc.) and how the user can best incorporate them into an overall healthy diet. The third level focuses on adopting healthy eating habits and behaviors (i.e., eating out, grocery shopping, frugality, commensality, etc.). Presented as an example of a HLS, the Mediterranean diet and lifestyle were previously encouraged in a nutritional intervention among Midwestern career firefighters in which many website resources have been reutilized (28, 29). Despite its cultural and geographic origins, the principles of the Mediterranean diet can be culturally translated and easily adapted in non-Mediterranean populations (30, 31). Furthermore, national firefighter surveys on dietary preferences showed that the Mediterranean diet consistently received highest favorability ratings (32, 33). Similar dietary patterns concerned with overall diet quality, such as the DASH and MIND diets, may also offer comparable health benefits in this population (34, 35). Additional resources included the “Nutrition Source” from the Harvard T.H. Chan School of Public Health (36), “Oldways” a non-profit organization dedicated to the dissemination of traditional cultural diets (37), and the National Institutes of Health (38).

#### 2.2.2 Physical activity domain

The objective of the Physical Activity domain is to encourage regular physical activity and help firefighters fine-tune their workouts so that they are well prepared for the physical demands of their job. The first level familiarizes firefighters with the core principles of physical activity (i.e., benefits, motivation, safety) and introduces them to the concept of “functional fitness.” The second and third levels expand on the “The Big Eight Method”, a firefighter functional fitness training regimen for optimal firefighter performance and longevity. With over 400 YouTube videos, this program was devised to provide firefighters with the tools, knowledge and mindset on how to optimize performance, reduce injuries/line-of-duty deaths, and extend career longevity. Firefighters learn how to easily program daily and weekly workouts, prioritize recovery and rest, as well as firefighter-specific hydration and nutrition (39). Each level contains exercises on functional strength, core strength, cardiovascular capacity, and flexibility that firefighters should implement into their daily workout routine. In addition, this domain includes materials from the Physical Activity Guidelines for Americans (40), as well as the Centers for Disease Control and Prevention’s webpage on physical activity (41).

#### 2.2.3 Sleep domain

The Sleep domain aims to impart knowledge and facilitate habitual sleep behaviors that can help firefighters, despite their irregular shift-work schedule, attain sufficient sleep quality and quantity and ward off fatigue. The first level revolves around the importance of sleep and the impact of insufficient sleep on firefighters. The second level focuses on how to implement good sleep hygiene. The third level focuses on tracking sleep, understanding sleep disorders, and effective ways to address them. The educational material within the sleep domain is based on the National Sleep Foundation’s online resources (42) and the Division of Sleep Medicine at Harvard Medical School (43), as well as other firefighter websites which offered a more firefighter-centered viewpoint on achieving better sleep.

#### 2.2.4 Resilience domain

The goal of the Resilience domain is to help firefighters cultivate the necessary “mental skillset” to deal with adversity and thrive in both their career and personal lives. The first level serves as an introduction to stress and basic relaxation techniques to help counteract negative consequences of stress. The second level moves on to resilience and self-care practices (i.e., gratitude journal, relaxation techniques, mindfulness) and learning how to deal with cognitive distortions. The third level focuses on the negative emotions and mental health issues that often affect firefighters. The resilience domain includes content inspired by the American Psychological Association (44), the National Institutes of Mental Health (45), and the Center for Wellness and Health Promotion at Harvard University (46). These general-purpose resources were complemented with firefighter-specific content including fire experts’ testimonies and perspectives on resilience and mental health.

## 3 Methods

### 3.1 Alpha and pre-beta testing of the MVP

**Figure 4:**
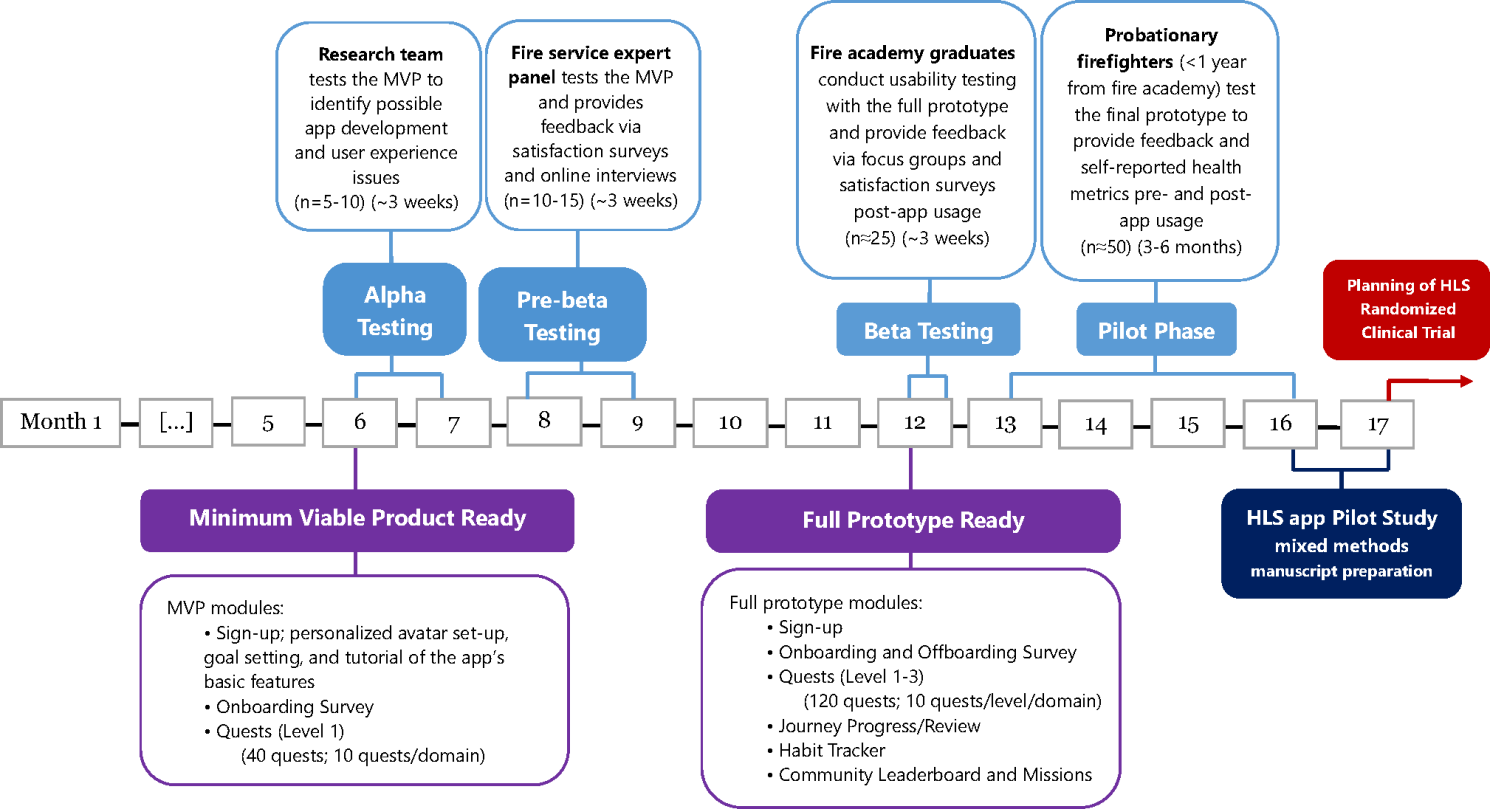
Flowchart diagram of the HLS app *Surviving & Thriving* development and pilot study design.

**Figure 4** shows the flowchart diagram of the HLS app *Surviving & Thriving* development and pilot study design. At this time, the first aim has already been carried out. The first stage of internal testing involved “alpha” testing, which was conducted by the research team (n=5-10) by simulating the firefighter user experience. Alpha testing was done to identify possible problems and development errors of the MVP before it is shared with users within the target population. The MVP included the full onboarding content, which consists of a validated short questionnaire for each of the four domains, personalized avatar set-up, goal-setting, and a tutorial of the app’s basic features. The research team and developers have refined the HLS app based on the feedback collected during alpha testing accordingly.

“Pre-beta” testing of the refined MVP involved our fire service expert panel associated with the research team (n=10-15). The participants were generally experienced firefighters or leaders at their fire departments. This was a more specific form of alpha testing because it was carried out by potential end-users or consumers of the app. However, given that the pre-beta users were affiliated with the research team, this form of testing is still considered a form of in-house acceptance testing by individuals who are aware and open that the product is under development. The purpose of pre-beta testing was to help improve its utility and functionality. The pre-beta participants were then asked to engage in a satisfaction survey and online interview to provide further feedback and suggestions on refining the content and usability of the newly developed app. This has allowed the research team to update the HLS app prototype before field testing the “pre-consumer” version.

### 3.2 Usability testing (beta testing)

By partnering with two academies in the New England area (Connecticut and Stow, MA) we will have access to over a hundred new firefighters to recruit from for “beta” testing. We will recruit ~55 new firefighters from recently graduated academy classes and ask that they complete 14 days of usability or beta testing on their smartphones. Our proposal has been designed to ensure that it is open to all probationary firefighters regardless of race or gender. The link to download the app will be emailed to the participants after they consent to participate and all participants are expected to explore the functions of the app. Although creating an account for the app requires using a personal email, the research team will not access any of the participants’ private information from the app, including email addresses. The data collected from the app (i.e. time spent on the app, screen views, retention, etc.) will be processed according to appropriate de-identifiable security procedures.

As part of beta testing, focus groups and satisfaction surveys will be carried out. The focus groups will be moderated by at least one experienced member from the research team. Focus group discussion guides have been adapted from existing literature on similar smartphone app usability testing (47). The participants are asked to verbalize what they think about, are looking at, doing, and feeling throughout the process of using the HLS app. Thereafter, members of the research team will independently analyze qualitative focus group data by coding the transcripts using commercial software such as NVivo 10 (QSR International, Victoria, Australia) to organize, sort and structure the data until saturation is reached and no new themes emerge. Similarly, a satisfaction survey adapted from a validated questionnaire will be distributed to the beta testing and Focus Group participants (n=30) (48).

### 3.3 Pilot study phase

This phase of the study aims to demonstrate that enrolled participants from the new firefighter study population can maintain adherence to the HLS app for at least 3-6 months. We will recruit ~50 new firefighters (a separate cohort from those enrolled in the beta testing) and monitor their app usage. App metrics such as session duration, screen views, and user retention within the app will provide insights into how users interact with the app over time. This cohort of new firefighters for the pilot study phase will also assess the HLS app’s ability to maintain/improve HLS parameters (HLS scores, physical fitness, mental health (trauma/depression) screeners). For the purposes of this pilot study, we will conduct a small single arm trial which aims to obtain preliminary evidence of the efficacy of a novel digital intervention and to collect qualitative feedback from a subgroup of target users. Changes in HLS parameters throughout the pilot testing will be evaluated with repeated measures of health metrics pre-and-post app usage among those participants that engaged in the HLS app for 3-6 months. Namely, participants will receive the same lifestyle and mental health questionnaires and fitness test at the two time points: before using the HLS app and at the subsequent follow-up (i.e. 3-6 months after). Both adherence and efficacy results will be adjusted for the potential influence of new firefighters’ demographic characteristics. Effect modification analysis will include fire crew, station and department cultures/policies (measured by the “Health Culture” score as mentioned below in 3.3.4.).

#### 3.3.1 Healthy lifestyle score, physical fitness, and mental health outcomes

##### 3.3.1.1 Healthy lifestyle score

Information will be collected using a questionnaire comprised of individually validated components via the Research Electronic Data Capture (REDCap) online survey software. These components include: tobacco use status, Mediterranean diet adherence screener (MEDAS) score (49, 50), physical activity level (51), sedentary behavior (measured in hours spent watching TV per week) and sleeping patterns (measured in daily sleeping hours and frequency of naps per week) (52).

The HLS score consists of seven dichotomously assessed items present in a healthy lifestyle (including: weight control [non-obese BMI], not smoking, Mediterranean diet adherence, regular physical activity, limited tv-watching, adequate sleeping, and napping). A value of 0 or 1 is assigned to each of the seven HLS components. Participants are assigned a value of 1 for each of the following: no smoking in the last 6 months, moderate-high physical activity (>16 METs-h/wk), high adherence to Mediterranean diet pattern (MEDAS ≥9 points) (50), BMI less than or equal to 30 kg/m^2^, time watching television (<2 h/d), adequate sleeping (7-8 h/d) and taking naps throughout the day (siesta or after lunch short nap); otherwise participants were assigned a value of 0. Total HLS scores range from 0 to 7 (with 7 reflecting the highest HLS and 0 the lowest) and has been previously associated with lower risk of prevalent hypertension and higher aerobic capacity among young firefighter recruits (9). Components of the HLS score (e.g. MEDAS score, sleep pattern, physical activity level, etc.) will also be assessed individually.

##### 3.3.1.2 Physical fitness

Physical fitness will be measured by push-up exercise capacity (19). Participants will be asked to perform 1-min pushups, which will be counted continuously until a participant is exhausted and breaks the cadence. The procedure will be remotely supervised via Google Meet or other virtual techniques by one of the research team members.

##### 3.3.1.3 Mental health

Depression/Trauma symptoms will also be assessed using the modified Beck Depression Inventory (BDI-PC) (53); Patient Health Questionnaire (PHQ-9) (54); and a modified version of Posttraumatic Stress Disorder Checklist (PCL-5) (55). Based on extensive field experience, shorter versions of each type of screener will be disseminated to reduce “questionnaire-fatigue”. Additionally, all suicide/self-harm items have been omitted from the BDI-PC and PCL-5 to avoid the difficulty of triaging “yes” answers for psychiatric referrals in the context of research, rather than clinical assessments.

### 3.4 Covariates

Socio-demographic characteristics (i.e., age, sex, educational level, marital status) as well as behavioral health information will be collected via the online survey. Working conditions & fire department culture/policies will be measured post- app usage by means of “health culture” scores (**Table 1**). These scores rate the extent to which a new firefighter perceives that his/her department and co-workers value and promote healthy behaviors. Because we are unaware of any such previously validated instrument, with the input and oversight from our fire service expert panel, we have modified a set of questions from the Fire service Organizational Culture of Safety (FOCUS) survey with the addition of some questions on health-promoting environments used in other occupational settings (56, 57).

**Table 1:** Data Collection during the Pilot Study Phase of the *Surviving & Thriving* app.

Study Measures	Pre-app usage: <1 year from Fire Academy graduation	Post-app usage: 3-6 months of app engagement
Healthy Lifestyle questionnaire	**X**	**X**
Physical Fitness Measure (i.e., 1-min pushups)	**X**	**X**
Mental Health: Depression/Trauma symptoms (BDI-PC, PHQ-9 and PCL-5)	**X**	**X**
“Health Cultures” scores: ratings of each fire station’s/fire department’s environments (working conditions, culture/policies) as to encouraging/discouraging healthy behaviors)	–	**X**

### 3.5 Participant incentives

Aim 1 was conducted on a volunteer basis by the research team and fire service expert panel with no formal compensation for the alpha and pre-beta testing of the development phase. For Aim 2 on usability (beta testing) among 50 enrolled new firefighters, we will conduct Focus Groups via Cambridge Health Alliance (CHA) Google Meet (expected n=25) compensated with a gift card, and satisfaction surveys (expected n=30) compensated by a gift card to get additional “end-user” feedback. For Aim 3, ~50 new firefighters will be recruited for pilot testing and their app usage will be monitored if they have completed more than three months of use (expected n=50). Upon completion of the pilot phase, participants will be given an additional gift card for a follow-up collection of health measures to compare against their baseline values.

### 3.6 Statistical analysis

All data will be collected using de-identified study codes. Initial data merging, cleaning, management and basic statistical analyses will be performed using appropriate statistical software such as R software (version 4.1.0) (58) and Stata version 17.0 (59). For Aim 2, mixed methods will be applied, in which quantitative data will be collected through a satisfaction survey and qualitative data via focus group interviews. The latter will be analyzed using the thematic analysis method (60). After the focus group transcripts have been coded, they will be re-read to ensure that all relevant extracts have been identified and coded against the most appropriate theme. The themes will be grouped into main themes and subthemes, which will be hierarchically organized to reflect their prevalence in the data.

Regarding quantitative data (i.e., satisfaction survey data from the beta testing and all data collected from the pilot phase), continuous characteristics that are normally distributed will be presented as the mean ± SD and comparison between pre- and post- app usage using the paired t-test (or the repeated measures analysis of variance (ANOVA) for more than two groups), whereas those with skewed distributions will be presented as the median and interquartile range, and compared using non-parametric techniques such as the paired Wilcoxon test for two groups or the Friedman test for more than two groups. Dichotomized or categorical characteristics will be described as a frequency (%) and pre- and post- values compared using McNemar’s test or the chi-square test of independence, as appropriate.

In the pilot study phase, Aim 3, adherence to the HLS app will be measured continuously as the total number of engagements with the software by each participant over the 3 to 6-month pilot study. This outcome will be supported by other prespecified app-usage metrics facilitated by the Managed IT Services provider (i.e. app user information; sign-ins, quest completion rate for each level, frequency of habit tracker use, and number of missions initiated). The primary outcome for validating the efficacy of the HLS app among the new firefighters’ will be the changes in the HLS score, comparing the participants’ pre- and post- app-usage scores (10). Using a behavior change measure (i.e. HLS score) as a primary outcome is justified in an intervention of short duration (11). As secondary outcomes, we will evaluate changes in 1-min pushups and mental health parameters. In addition, we will examine whether the health culture in various fire service workplace environments, as measured by the Health Culture score, influences the intervention effect of the app on the primary and secondary outcomes.

Generalized linear models will be utilized to assess the effect of the proposed app intervention comparing pre and post HLS scores, mental health scores, and 1-min push-up capacity upon completion of 3-6 months of app usage. All models will have their assumptions verified, using analysis of the residuals and other diagnostic testing. Generalized linear modeling will be used to model changes over time after taking into consideration the specific variance-covariance structure of repeated measurements or using other statistical methods such as generalized estimating equations models. All models will be adjusted for demographic and working conditions and other potential confounders. Interaction terms will also be incorporated into the models to evaluate any potential effect modification by working conditions, cultures, and policies. Statistical significance for all analyses will be considered as a two-tailed *p*<0.05.

### 3.7 Ethics approval and data management

All protocols, questionnaires and procedures have been approved by the CHA Institutional Review Board and has received approval by the Compliance Assurance Program Office (CAPO) from the Department of Homeland Security. The IRB approved research will not begin without authorization from the CHA Office for Sponsored Research. All research activities will meet HIPAA Privacy and Security Rule and all members of the study team will comply to internal and external auditing by CHA Quality Assurance/Quality Improvement personnel. Signed consent forms and all data collection will be extracted and transferred to CHA password-protected internal servers and maintained confidentially. All electronic data will be restricted to authorized research personnel and will be password protected ensuring a low risk of breaching confidentiality. The study team will keep the key to the code in a password-protected computer and locked up for at least 7 years after study closure per OHRA’s Record Retention Policy. The research team will regularly review the data collected for the research project at least once a month to ensure data integrity throughout the duration of the research project.

### 3.8 Dissemination

The findings of the current project will be disseminated through various approaches including but not limited to publications, media, conference and meeting presentations to national and international audiences, such as the New England College of Occupational & Environmental Medicine (NECOEM), the American College of Occupational & Environmental Medicine (ACOEM). In addition, the findings will be disseminated directly to the fire service including presentations at the U.S. National Fire Academy in collaboration with the National Fallen Firefighters Foundation (NFFF), the International Association of Fire Fighters (IAFF) and the International Association of Fire Chiefs (IAFC)/Safety Health and Survival Section (SHS).

## 4 Discussion

This study protocol presents the proposed usability testing and piloting of a viable, user-friendly, healthy lifestyle app as a digital intervention tool for lifestyle behavior change among new, probationary firefighters in the US. Upon completion of the development of a full prototype overseen by the research team, the usability testing in the study target population will be followed by a satisfaction survey and focus groups, whereas the pilot study phase will observe the plausible changes in health behaviors to assess the feasibility of this novel mobile-app intervention.

Lifestyle-related diseases account for 2/3 of lifetime mortality among US firefighters, which can be largely attributed to major chronic disease risk factors, including unhealthy lifestyle behaviors such as tobacco, poor dietary quality, and physical inactivity (13). Notably, unhealthy dietary habits, overweight and obesity are prevalent among firefighters (3). Studies have shown that over 70% of US firefighters are either overweight or obese, exceeding the national average for adults in the US (61, 62) Additionally, hypertension, metabolic syndrome, dyslipidemia, and smokeless tobacco are also prevalent among US firefighters (63).

Recently, the occupational exposure as a firefighter was classified as carcinogenic to humans (Group 1) (64). While firefighters experience well-documented carcinogenic exposures (65, 66), there is substantial evidence that cancer risks are reduced by following healthy diets, engaging in regular physical activity, and avoiding obesity (67, 68). Similarly, other growing areas of concern regarding firefighters health include the negative consequences of repeated exposure to traumatic events that may lead to depression, post-traumatic stress, substance abuse, and suicide (3, 69, 70). There is significant evidence and data supporting that healthy behaviors reduce the risks of and can improve symptoms of depression and other mental health disorders. These healthy behaviors include adherence to a healthy diet (71), engaging in regular physical activity (72), avoiding excessive sedentary behavior (73), and a higher adherence to an overall HLS (11, 74). Thus, this study plays a role in occupational health care by overseeing the development of a digital tool for a comprehensive workplace health promotion and occupational health strategy with scientific vigor.

Despite existing evidence on the positive effects of good “safety” cultures in the fire service, studies are limited on health-promoting climates and firefighter health (56, 75). Evidence from our earlier case-control and national-level studies established the interaction of work duties with lifestyle among firefighters and underlying diseases associated with on-duty cardiac events (2, 5, 76). Yet, their risks can all be mitigated by better nutrition and other lifestyle measures (32). Our preliminary work has indicated the necessity and importance of a user-friendly HLS app intervention well suited to the new firefighters for better maintenance and improvement of HLS parameters (11).

To the best of our knowledge, this is the first HLS app that specifically addresses new firefighters’ health and well-being. Moreover, unlike most HLS apps who focus only on one or two lifestyle aspects, our app takes a more holistic approach and thoroughly covers four of the most essential HLS domains (nutrition, sleep, physical activity, resilience) (77–80). Thorough knowledge of the target population is instrumental to an app-based intervention’s success, therefore, *Surviving and Thriving* will be occupationally-tailored to new US firefighters. There is an important gap in the digital health movement to meet this particular workforce’s lifestyle needs, to not only survive but also thrive, in light of the demands of their career.

The creation of the app prototype was preceded by extensive literature reviews and even a qualitative study by our research team in order to investigate how to best accommodate the needs of our target population and how to best address potential facilitators and barriers to adherence to an HLS intervention (26). The educational content of the app was mainly derived from firefighter-specific resources and the graphics for the wireframes were designed to be firefighter-themed, where applicable. We also made sure that the daily workload associated with the app would be flexible enough to incur behavioral changes but not interfere with the work demands and busy lifestyle of professional firefighters. Most importantly, once pre-beta testing has been completed, the app will be evaluated by focus groups consisting of experienced firefighters at first and new firefighters after beta testing. Their feedback and ideas will then be used to optimize the app prototype before the pilot study phase and to ensure that the final version fits the end-users’ needs.

In order to counteract the high attrition and low long-term engagement rates traditionally associated with app-based interventions, we have employed multiple gamification strategies, a scoring system *(surviving* and *thriving* points*)* as well as the inclusion of a habit tracker and community missions (81). Other aspects targeting user engagement include goal setting, notifications that reinforce healthy behaviors, a leaderboard to compare users’ progress, levels and incremental challenges for the individual, team-based challenges, and allocation of points, coins, and badges for milestones) which have been shown to incentivize engagement, foster behavioral changes more effectively and make the use of an app more appealing (82, 83).

We recognize that our study and app prototype have some potential limitations. First, a potential response bias is dependent on quest completion rates and overall evaluations of individual performance and progress, which greatly rely on what the user reports and/or uploads, which may not be congruent with their actual behavior. Second, participants will be aware of their participation in a pilot study of a lifestyle behavior change tool and the fact that they are under observation, our pilot study will probably be susceptible to performance bias and social desirability. Third, it can be argued that the pilot phase, which spans 3 to 6 months, may be too short to gauge whether the app-based intervention will have a significant impact on the health outcomes of professional firefighters, whose careers are usually decades-long. Regarding the technical features of the app itself, at this stage there is no option to integrate data from other mobile apps that estimate a range of cardiometabolic and other health parameters that the user may be already using. Also, we recognize that there is limited personalization since all users will have the same set of tasks that are required to complete the avatar’s journey. Nonetheless, the order of their weekly quests/tasks will be arranged based on their baseline goal setting.

Ultimately, our goal is to develop and test the usability and feasibility of a viable, user-friendly and engaging HLS app that will help new firefighters maintain and improve their training-derived health/fitness levels more effectively, as compared to those before engaging with the HLS app. Firefighters that engage regularly with the app are expected to demonstrate measurable and plausible changes in the HLS parameters covered by the app (nutrition, sleep, physical activity, resilience) in the desired direction. The current research will support the implementation of this app as the primary intervention strategy in a multi-center controlled trial in the US fire service. The proposed future randomized clinical trial would collect laboratory tests to study the potential effect of this digital intervention on chronic disease risk factors in addition to habitual lifestyle behaviors. Ultimately, by sustaining the academy/training-derived health benefits into the probationary firefighter period, we expect firefighters will be more likely to maintain good health habits throughout their careers and thus increase their career longevity.

## Author contributions

Conceptualization or design of the project: XC, F-YL, SK. Conceptualization or design of the app content: MH, EB, IL, YH, TF, JG, AG. Writing-original draft preparation: MH, EB, KH, YH, F-YL. Writing-review and editing critically for important intellectual content: MH, AG, F-YL, MS-P, SK. Visualization: XC, MH, F-YL, EB. Supervision and project administration: F-YL, SK. Funding acquisition: SK. F-YL, MS-P, MH, IL, KH, and SK have full access to this study and take complete responsibility for the integrity of this project. All authors have read and given final approval of this version to be published.
